# Abdominal Wall Postherpetic Pseudohernia

**DOI:** 10.5826/dpc.1101a96

**Published:** 2020-12-10

**Authors:** Gerald Selda-Enriquez, Ana Laura Melian-Olivera, Patricia Burgos-Blasco, Daniel Ortega-Quijano

**Affiliations:** 1 Servicio de Dermatología, Hospital Universitario Ramón y Cajal, IRYCIS, Madrid, Spain

**Keywords:** postherpetic pseudohernia, herpes zoster, infectious disease, dermatology

## Case Presentation

**Figure 1:**
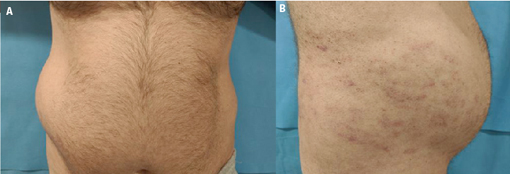
(A) Bulging is seen in the right abdomen. (B) Erythematous maculopapular lesions due to herpes zoster are observed along the right T9–10 dermatome, consistent with the area where bulging is seen.

An otherwise healthy 49-year-old man presented to the emergency department with complaint of a 3-day history of abdominal bulging (Figure 1A) in the same location of a previous herpes zoster infection. The bulge increased with Valsalva maneuver. Skin examination still revealed residual lesions of herpes zoster (Figure 1B). The final diagnosis was postherpetic pseudohernia, and at the 3-month follow-up visit, it had disappeared.

## Teaching Point

Herpes zoster is characterized by clustered maculopapular and vesicular lesions along a dermatome as a result of reactivation of varicella zoster virus in the dorsal root ganglia of peripheral nerves. An infrequent complication is postherpetic pseudohernia which arises when the anterior root ganglia is involved, thus provoking muscular paralysis [1]. Differential diagnosis with true hernia by means of clinical and radiological exams is critical, as it does not require surgery and tends to disappear spontaneously in the majority of cases after less than a year [2].
